# Exposure reduction in COVID-19 autopsies

**DOI:** 10.4322/acr.2020.193

**Published:** 2020-07-23

**Authors:** Christopher Siefring, Jennifer Sachire, Diana Thomas, Patricia Allenby

**Affiliations:** a Ohio State University, Department of Pathology, Regional Autopsy Center. Columbus, OH, United States of America.

**Keywords:** Personal protective equipment, surface decontamination

## DEAR EDITOR

**Figure 1:**
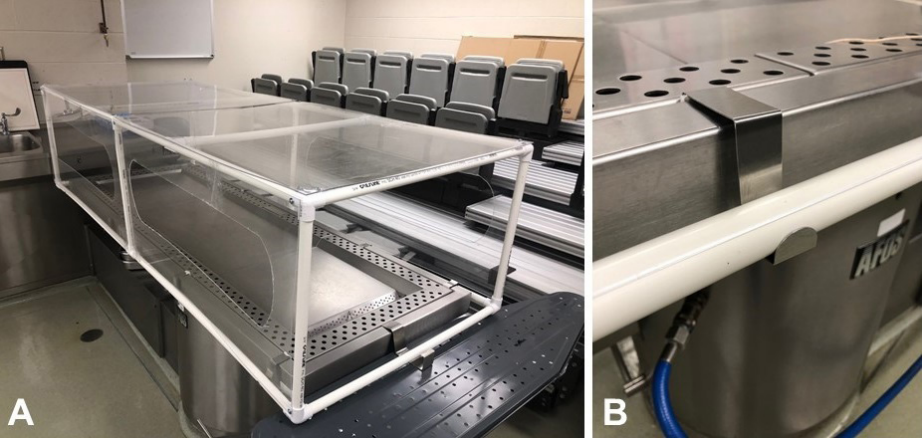
R.A.G.E. cage full set up with ironing board support (**A**) and close up of suspension with door hooks (**B**).

With personal protective equipment (PPE) in high demand during the COVID-19 pandemic, members of the clinical and laboratory communities, including those in autopsy pathology, have had to conserve PPE and revise protocols to expand safety measures. Many institutions are not performing autopsies on COVID-19 patients due to lack of PPE and appropriate environmental controls. Others are performing limited autopsy examinations to decrease risk to staff. Until the infectiousness of SARS-CoV-2 in postmortem tissues and fluids can be quantified, the U.S. Centers for Disease Control and Prevention and The College of American Pathologists (CAP) recommends standard, contact, and airborne precautions to be practiced during autopsies on COVID-19 positive patients, as well as limiting aerosol-generating procedures.^1^ ^,^ ^2^ While these precautions are important and effective, splash risk and surface contamination of tissues during handling are still prevalent during these procedures. To help mitigate splash risk, potential aerosolization, and surface contamination, two specialty devices were built to decrease exposure to contaminated fluids and materials (Figure 1).

The first device, named the R.A.G.E. (Reduction in Autopsy Grossing Exposure) cage, was developed to supplement protection to the autopsy staff in addition to PPE and environmental controls. Engineering and fabrication means were limited, so design and construction were kept to feasible methods with standard hand and power tools. The cage consists of readily available 3/4 inch PVC pipe, acrylic glass panes, standard metal screws, and hardware (approximate cost $300). The PVC pipe and acrylic panes were cut to size at local hardware stores and the pieces assembled by the autopsy staff. “Hand slots” for access were cut into the acrylic using a standard rotary tool. Once aligned, pilot holes were drilled into the acrylic and PVC and were screwed together. The cage is slightly larger than the dimensions of the autopsy table to allow full coverage. Suspension was achieved using over-the-door hooks wedged into the perimeter of the table. For extra support at the head of the table, an adjustable ironing board was placed underneath (Figure 1).

In addition to the cage, a two-person autopsy performance protocol was developed to decrease splash leading to potential aerosolization. This procedure utilizes an assembly line workflow with personnel alternating between “cutting” and “measurement/handling” roles. Our experience with the R.A.G.E. cage has proven to decrease excessive splash during fluid suctioning and organ removal. While all staff wears powered, air-purifying respirators (PAPR) with full face coverage, the R.A.G.E. cage has prevented incidents that otherwise would have led to staff and autopsy suite contamination. This protection does come at a small cost of mobility during autopsy performance, but with the two-person protocol mentioned above, this is minimal. R.A.G.E. cage materials can be easily wiped, disinfected, and stored after the conclusion of the case.

**Figure 2:**
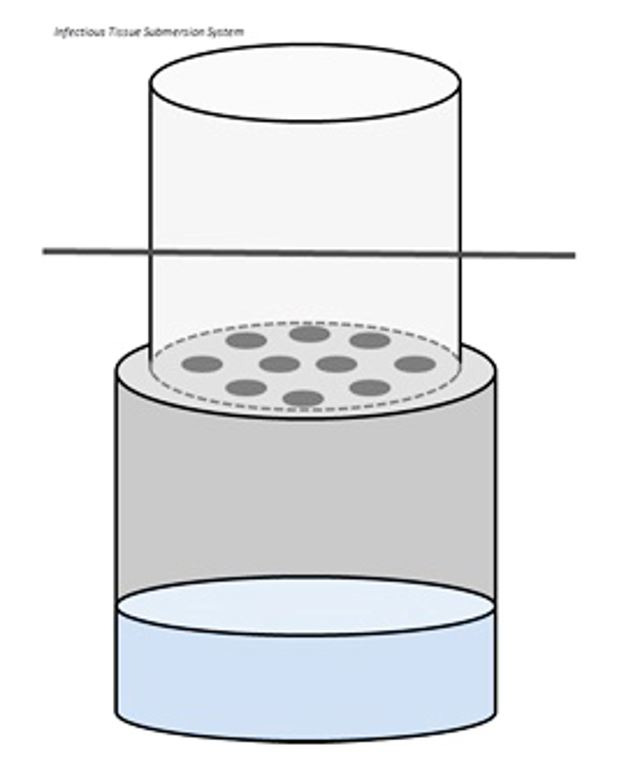
D.U.N.K. tank schematic.

The second device that was developed is called the “viral Deactivation Under Nesting-compression Kinetics” (D.U.N.K.) tank. The D.U.N.K. tank is a simple device composed of two nesting plastic buckets. One is filled approximately halfway with 10% formalin, and the second is perforated with several coin-sized holes on the bottom (Figure 2).

Organs of interest that pose higher a risk of viral contamination are placed into the formalin bucket and are then compressed with the perforated bucket. Compression increases the likelihood that the entirety of the organ(s) external surface is decontaminated. In cases of patients diagnosed with COVID-19, the heart and lungs are placed in the D.U.N.K. tank and decontaminated for 10 minutes.^3^ ^,^ ^4^ Further insufflation of the lungs and stuffing of the heart chambers with formalin-soaked gauze may be accomplished safely.

It is worth considering that while the COVID-19 pandemic is expected to subside in the upcoming months, the current challenges have increased personal and institutional awareness on resource limitations. The responsibility lies on the healthcare industry to work together to conserve resources and innovate new solutions that can reinforce the abilities to provide optimal patient care while maintaining safe working conditions.
